# Fiber-based food packaging materials in view of bacterial growth and survival capacities

**DOI:** 10.3389/fmicb.2023.1099906

**Published:** 2023-01-26

**Authors:** Paul Jakob Schmid, Stephanie Maitz, Nadine Plank, Elisabeth Knaipp, Sabine Pölzl, Clemens Kittinger

**Affiliations:** Diagnostic and Research Institute of Hygiene, Microbiology and Environmental Medicine, Diagnostic and Research Center for Molecular Biomedicine, Medical University of Graz, Graz, Austria

**Keywords:** food packaging, food contamination, bacterial growth, packaging material, cellulose, survival, endospores

## Abstract

Understanding interactions of bacteria with fiber-based packaging materials is fundamental for appropriate food packaging. We propose a laboratory model to evaluate microbial growth and survival in liquid media solely consisting of packaging materials with different fiber types. We evaluated food contaminating species (*Escherichia coli*, *Staphylococcus aureus*, *Bacillus cereus*), two packaging material isolates and bacterial endospores for their growth abilities. Growth capacities differed substantially between the samples as well as between bacterial strains. Growth and survival were strongest for the packaging material entirely made of recycled fibers (secondary food packaging) with up to 10.8 log_10_ CFU/ml for the packaging isolates. Among the food contaminating species, *B. cereus* and *E. coli* could grow in the sample of entirely recycled fibers with maxima of 6.1 log_10_ and 8.6 log_10_ CFU/mL, respectively. *Escherichia coli* was the only species that was able to grow in bleached fresh fibers up to 7.0 log_10_ CFU/mL. *Staphylococcus aureus* perished in all samples and was undetectable after 1–6 days after inoculation, depending on the sample. The packaging material strains were isolated from recycled fibers and could grow only in samples containing recycled fibers, indicating an adaption to this environment. Spores germinated only in the completely recycled sample. Additionally, microbial digestion of cellulose and xylan might not be a crucial factor for growth. This is the first study describing bacterial growth in food packaging materials itself and proposing functionalization strategies toward active food packaging through pH-lowering.

## 1. Introduction

One essential function of food packaging is to protect food from chemical, physical or microbiological changes to extend the shelf life and thereby ensure the quality of the food as well as preserve the consumer’s health. In recent years, extensive research on active food packaging has aimed to provide new materials to maintain microbial food integrity beyond traditional packaging systems (Norrrahim et al., 2021). However, many active food packaging strategies are still in their infancy, demand complex functionalization and therefore can only address single steps in the food supply chain (Azevedo et al., 2022). In contrast, the sources of microbial contaminants are manifold and may occur in all steps of food production, transportation, storage and preparation. A large number of different packaging materials are already used in this field to meet the requirements of product quality and safety. However, these established materials are often poorly investigated from the aspect of active food packaging. Fiber-based packaging materials stand out as cost-effective, biodegradable and sustainable solution in the food packaging field due to renewable raw materials with a large degree of recycling possibilities. Furthermore, a huge diversity of different fiber types and a variety of formulations allow the production of packaging materials for any kind of application. Moreover, a microbiological superiority of fiber-based food packaging materials against plastic packaging has been shown more than once (Patrignani et al., 2016; Siroli et al., 2017). The beneficial effect of fiber-based packaging materials on the cross-contamination potential for stored food has been explained by the capability of entrapping microorganisms within the fiber-network together with a faster loss of viability compared to plastic surfaces (Siroli et al., 2017), indicating active food packaging properties. Nevertheless, little is known about the interactions of microorganisms with the surrounding matrix of fiber packaging due to the high diversity of different types of packaging. While factors affecting a potential transfer of bacteria to contact surfaces have already been assessed (Maitz et al., 2022), the knowledge about microbial growth and survival in food packaging materials needs to be extended. Previous research on the interactions of bacteria with fiber-based packaging materials has largely dealt with fermentation strategies to produce energy carriers such as hydrogen and methane (Ntaikou et al., 2009; Brummer et al., 2014; Asato et al., 2016; Poladyan et al., 2020), or bioplastics (Abdelmalek et al., 2022). These studies usually aimed to optimize waste treatment and used packaging material waste, which was physiochemically treated prior to microbial digestion. Other research focused on spatial interactions and therefore growth media were supplemented to promote microbial growth at the expense of assessing growth capacities (Suominen et al., 1997; Hol et al., 2019). Numerous studies have been conducted on microbial growth in packaged foods, but to our knowledge, no study has ever examined bacterial growth capacities in the food packaging material itself, although fiber-based packaging materials have been postulated to provide a thriving environment for bacteria (Brandwein et al., 2016). More knowledge in this field may contribute to improved types and applications of fiber-based packaging in the food sector as well as point out novel strategies for functionalization. Therefore, this research aimed to evaluate the growth capacities of certain bacterial species in four artificial growth media using only fiber-based packaging materials as a basis, differing in recycled fiber content and fiber bleaching. The tested bacterial strains are related to various food packaging issues either by representing species likely responsible for cross-contamination events, common food contaminants or packaging-inherent microorganisms. *Escherichia coli* (*E. coli*) and *Staphylococcus aureus* (*S. aureus*) are two examples of food-contaminating species responsible for several severe food-borne outbreaks in the last decades (Hennekinne et al., 2012; Yang et al., 2017). The presence of bacteria in fiber-based packaging materials has been intensively studied covering the raw materials, the manufacturing environment and the final product (Väisänen et al., 1998; Zumsteg et al., 2017). Studies on packaging materials revealed a predominance of Gram-positive, mesophilic, endospore-forming bacteria, mostly belonging to the family of Bacillaceae (Suihko et al., 2004; Lalande et al., 2014), including food relevant *Bacillus cereus* (*B. cereus*) (Schmid et al., 2021). Within the last years, the COVID-19 pandemic has drawn public attention to possible cross-contamination events through packaging materials (De Oliveira et al., 2021). However, this cross-contamination involves not only virus particles, but also bacteria in particular when it comes to food contamination. Although hardly any risk for food contamination emanates from the packaging inherent bacteria (Ekman et al., 2009), two members of the Bacillaceae were isolated from a packaging material sample for a more comprehensive study. Furthermore, spore suspensions of *Bacillus subtilis* (*B. subtilis*) and *B. cereus* were tested for their germination and growth capabilities. Both, *B*. *subtilis* and *B*. *cereus* are also considered as food spoilage agents (Stenfors Arnesen et al., 2008; Moschonas et al., 2021), especially the latter being known for causing foodborne infections and intoxications. In addition, we investigated factors that can promote or preclude growth, survival and decrease of microorganisms in fiber-based packaging materials by examining cellulose- and xylan-degrading properties of the microorganisms tested and evaluating the role of the pH as intrinsic antimicrobial factor of different packaging materials.

## 2. Materials and methods

### 2.1. Samples

In this study, four different cellulose-based fiber materials (PM 1–4) manufactured from three European packaging facilities were included. The samples were provided by industrial partners and were taken by instructed workers after manufacturing, wrapped in aluminum foil and sent to laboratory in sealed plastic bags. All samples came from the food packaging sector with different applications as defined by the manufacturer (Table 1).

**Table 1:** Packaging material samples.

Sample	Fiber type	Packaging type	Application
PM 1	Fresh fibers, bleached	Primary food packaging	Direct food contact (dry/moist/fatty)
PM 2	50% recycled fibers	Primary food packaging	Direct food contact (dry/moist/fatty) direct contact with food products that must be peeled or washed before consumption
PM 3	100% recycled fibers	Secondary food packaging	No direct food contact
PM 4	Fresh fibers, unbleached	Primary food packaging	Direct food contact (dry/moist/fatty)

Samples and corresponding information about the fiber type, food packaging type and application according to the manufacturers’ specifications.

### 2.2. Bacterial strains

The bacterial reference strains used in this study are listed in Table 2. Additionally, a spore suspension of *B. cereus* DSM 345 was prepared using sporulation agar according to EN ISO 7932:2004/prA1:2018 (International Organization for Standardization, 2020). After incubation at 30°C for 5 days, the spores were harvested with 5 ml Sörensen’s Phosphate Buffer including KH_2_PO_4_ (0.067 mol/l, Merck KGaA, Darmstadt, Germany) and Na_2_HPO_4_ · 2 H_2_O (0.067 mol/l, Merck KGaA) adjusted to a pH of 7. Afterwards, the spore suspension was centrifuged (4,200 rpm, 15 min, 4°C) and the pellet was washed with Sörensen’s Phosphate Buffer up to four times until high purity of spores was verified using a light microscope. The spore suspension was finally stored in ddH_2_O at −80°C. In order to investigate growth capacities and characteristics of packaging material inherent bacteria, two bacterial strains (strain 3.1, strain 3.2) were isolated from PM 3, identified on species level by MALDI-TOF (VITEK® MS, bioMérieux Marcy-l’Étoile, France) and 16S rRNA gene sequencing and included in all experiments. In brief, one gram of packaging material was disintegrated with 99 ml of 0.9% saline solution in sterile plastic bags using a Bagmixer (Interscience, St. Nom la Bretèche, France), followed by plating 500 μl on tryptic soy agar (TSA, Oxoid Deutschland GmbH, Wesel, Germany) and incubation at 37°C for 24 h. Two different colonies were randomly picked. For the species identification, strains were grown on COL-S blood agar (Becton Dickinson Austria GmbH, Vienna, Austria) at 37°C overnight, followed by MALDI-TOF identification and 16S rRNA gene sequencing using primer pairs 8FPLm and 806R for the 5′ fragment of the 16S rRNA gene (Relman, 1993). The INVISORB Spin DNA Extraction Kit (Invitek Molecular, Berlin, Germany) was used for DNA purification and amplification products were sent to Eurofins Genomics Germany GmbH (Ebersberg, Germany) for sequencing. Species affiliation was performed using the BLAST Sequence Analysis Tool (Altschul et al., 1990).

**Table 2:** Bacterial reference strains used in this study.

Bacterial strain	Origin
*Escherichia coli* DSM 1576	German Collection of Microorganisms and Cell Cultures GmbH (DSMZ), Braunschweig, Germany
*Staphylococcus aureus* DSM 799	DSMZ, Germany
*Bacillus cereus* DSM 345	DSMZ, Germany
*Bacillus subtilis* (BGA) spore suspension	Merck KGaA, Darmstadt, Germany
*Cellulomonas uda* DSM 20108	DSMZ, Germany

### 2.3. Growth and survival experiments

For preparation of media used to investigate microbial growth, one gram of a packaging material sample was evenly homogenized with 99 ml of 0.9% saline solution in sterile plastic bags using a Bagmixer. Subsequently, the fiber suspension was transferred into an Erlenmeyer flask and sterilized by autoclaving at 121°C. For pH measurements, 10 ml of the packaging material medium were centrifuged at 4,200 rpm for 15 min followed by pH determination in the supernatant using a pH meter (Orion 3 Star, Fisher Scientific (Austria) GmbH, Vienna, Austria). To study the influence of pH in PM 4, the 0.9% saline solution was replaced with phosphate buffered saline (PBS, Merck KGaA) in all steps of the experiment. The sterile packaging material media were inoculated with one of the following species: *E. coli*, *S. aureus*, *B. cereus*, as well as the two packaging material isolates. For this purpose, overnight cultures were grown in tryptic soy broth (TSB, Oxoid Deutschland GmbH) at 30°C (*B. cereus* and the isolated strains) or at 37°C (*E. coli* and *S. aureus*). Afterwards, the optical density at 600 nm (OD_600_) of the bacterial suspension was measured, followed by three washing steps in sterile 0.9% saline solution and centrifugation at 4,200 rpm for 15 min each to remove remaining traces of TSB. Then, the bacterial suspension was adjusted to an OD_600_ of 0.5 in sterile 0.9% saline solution. Generally, a bacterial suspension with OD_600_ of 0.5 equals approximately 8 log_10_ CFU/mL including species and strain variations. To study both eventual growth and survival, packaging material media were inoculated with bacterial cells in order to reach a final cell density of approximately 1 to 3 log_10_ colony forming units (CFU) /mL and 5 to 7 log_10_ CFU/mL, respectively, in the culture flask. All dilutions were performed with 0.9% sterile saline solution. After inoculation, the flasks were shaken at 120 rpm for 5 min in order to ensure even distribution of the spiked-in bacteria, immediately followed by initial determination of the microbial count and verification of the inoculum size. To determine the bacterial count, pour plate method was performed in which 1 ml was transferred either directly from the culture flask or appropriately diluted into a sterile petri dish and covered with 15 ml warm TSA. Bacterial counts were performed in technical triplicates. Agar plates were incubated at 30 or 37°C for 24 h and CFU were counted. The culture flasks were then incubated at 30 to 37°C with 50 rpm shaking. Further determinations of the bacterial count were performed after 1, 2, 3, 6, and 7 days of incubation. In addition, we inoculated the packaging material media with bacterial spore suspensions of *B. cereus* DSM 345 and *B. subtilis* subsp. *spizizenii* according to the protocol described before. All experiments were performed in biological duplicates.

As a contamination control, sterile medium was included for each packaging material medium to verify proper autoclaving. After each growth experiment, the correct presence of inoculated species was verified by streaking the packaging medium on COL-S agar and subsequent MALDI-TOF identification. Spore germination was assessed by selected microscopic examination of the growth media. For this purpose, 5 to 10 ml of culture medium were pelleted and microscopically examined with the Zeiss Axio Lab.A1 microscope (Carl Zeiss AG, Oberkochen, Germany) and image capture software ZEN version 3.2 (Carl Zeiss AG).

The data was analyzed and visualized in GraphPad Prism version 7.0.0 for Windows (GraphPad Software, San Diego, United States).

### 2.4. Determination of cellulose- and xylan-digestion abilities

To test potential cellulose digestion by the tested bacteria, we evaluated two carboxymethyl cellulose (CMC) media for CMC-assays previously described (Amore et al., 2013). For the CMC medium A, 5 g sodium CMC, (Sigma-Aldrich, Merck KGaA), 5 g Bacto™ tryptone (Thermo Fisher Scientific, Vienna, Austria) 4 ml micro salt solution consisting of 46.12 μl H_3_PO_4_ (Merck KGaA), 11.12 mg FeSO_4_ (Merck KGaA), 5.94 mg ZnSO_4_ (Merck KGaA), 0.436 mg CuSO_4_ (Merck KGaA), 2.5 mg MnSO_4_ (Merck KGaA), 0.6 mg Co(NO_3_)_2_ (Merck KGaA), 0.6 mg Na_2_MoO_4_ (Merck KGaA) and 1.24 mg H_3_BO_3_ (Merck KGaA) in 1000 ml dH_2_O, as well as 5 g NaCl (Carl Roth GmbH +Co. KG, Karlsruhe, Germany), 2 g (NH_4_)H_2_PO_4_ (Merck KGaA), and 15 g agar-agar (Carl Roth GmbH +Co. KG) were dissolved in 1 l deionized water by heating to 60°C, succeeded by autoclaving and agar plate preparation. For CMC medium B, 0.02% Remazol Brilliant Blue R (Merck KGaA) were added after autoclaving. Remazol Brilliant Blue R was intended for visualization of cellulose and xylan degradation indicated by a halo zone around colonies as mentioned by Amore et al. (2013). TSA was supplemented with 5% CMC as an additional medium, in case CMC medium A and B did not result in growth. All bacterial strains included in this study were grown on TSA and then streaked on CMC medium A and B and incubated at 30°C for 4 days. If colonies were found, they were picked and a bacterial suspension equal to McFarland standard 0.5 was prepared and diluted 10^−3^. Then one μL was spotted on CMC medium A and incubated for another 4 days at 30°C. After incubation, the CMC medium A was stained by flooding the agar plate with 0.1% aqueous Congo red solution (Carl Roth GmbH +Co. KG) for 10 min and subsequent two times washing with 5 M NaCl to visualize cellulose degradation indicated by a clear halo around the colonies (Amore et al., 2015). Halo zones were measured by taking the total diameter including colony and halo minus the diameter of the colony. All strains were tested in triplicates. For xylan digestion assay, 1,000 ml ready-to-use TSA base were prepared according to manufacturer’s protocol and supplemented with 5 g of birch wood xylan (Carl Roth GmbH +Co. KG) before autoclaving and agar plate preparation. All bacterial strains were pre-grown on TSA, then a bacterial suspension equal to McFarland 0.5 was prepared and one μL was spotted on xylan medium and incubated at 30°C for 4 days. Afterwards, the agar plates were stained with 0.1% Congo red and washed with 5 M NaCl. If possible, halo diameters on xylan plates were measured as for CMC plates. As a control, bacterial strains were grown in medium A without CMC and TSA and then stained.

## 3. Results

### 3.1. Growth and survival studies on bacterial food contaminants in packaging material media

**Figure 1:**
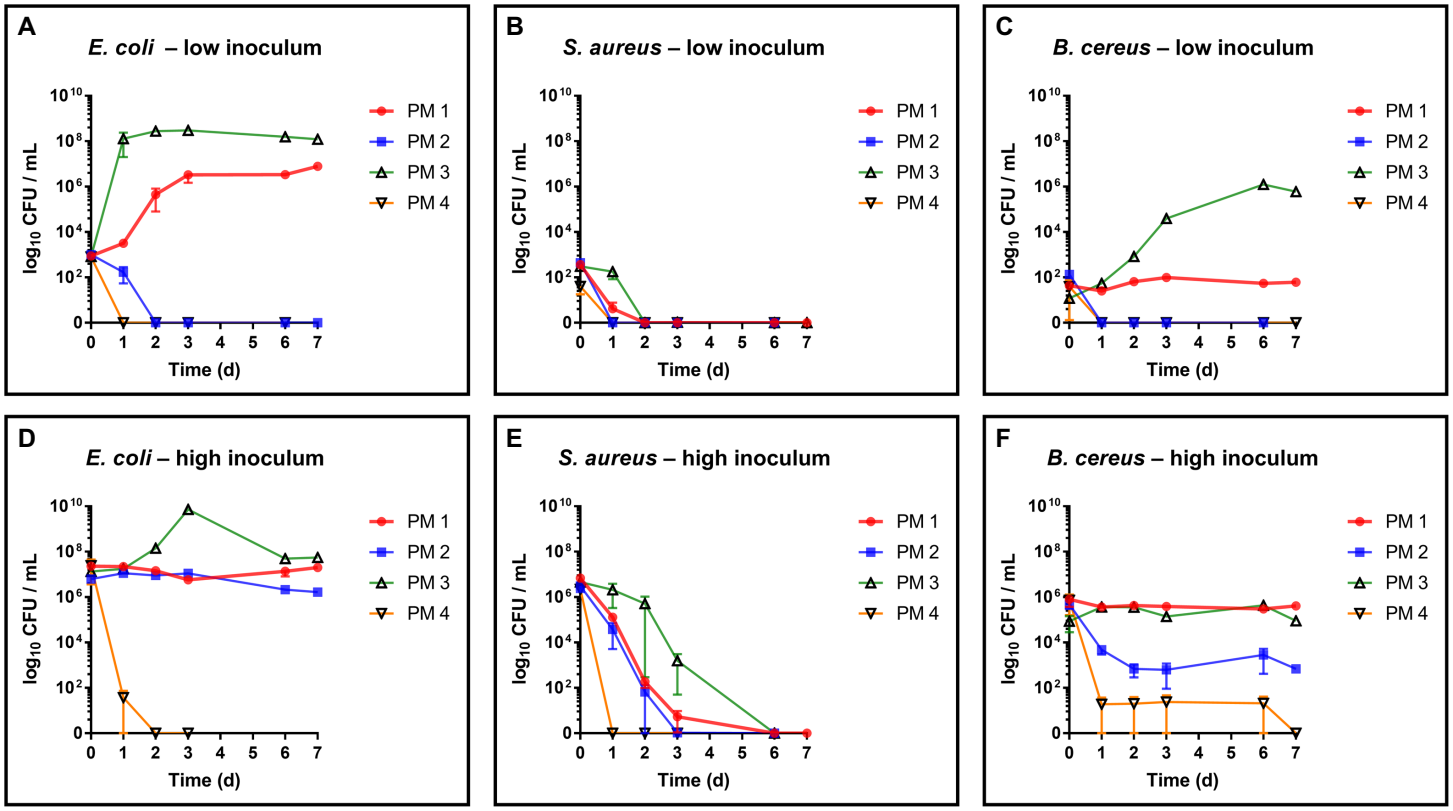
Evaluation of growth and survival of food contaminating species. Growth and survival were evaluated over time in liquid media solely consisting homogenized packaging materials. PM 1 (red) consists of bleached fresh fibers, PM 2 (blue) consists of 50% recycled fibers, PM 3 (green) consists of 100% recycled fibers, and PM 4 (yellow) consists of unbleached fresh fibers. **(A)** *E. coli*, low inoculum (1 to 3 log_10_ CFU/mL), **(B)** *S. aureus*, low inoculum, **(C)** *B. cereus*; low inoculum, **(D)** *E. coli*, high inoculum (5 to 7 log_10_ CFU/mL), **(E)** *S. aureus*, low inoculum, and **(F)** *B. cereus* high inoculum. All experiments were performed in biological duplicates. The mean with range is plotted.

The critical food contaminants *E. coli*, *S. aureus* and *B. cereus* were spiked in four different sterilized and homogenized packaging material samples to evaluate their growth and survival capacities in this environment. Monitoring over 7 days of the spiked growth media revealed distinct differences in the growth capacities of the test strains depending on the type of fiber-based packaging material used (Figure 1). Microbial growth and survival was primarily associated with the 100% recycled fibers medium and the bleached fresh fibers medium, whereas the media composed of 50% recycled fibers and unbleached fresh fibers, respectively, resulted in both, lower microbial survival and no observable growth. For growth evaluation, the above listed bacterial species were added to the samples in quantities leading to bacterial counts ranging from 1 to 3 log_10_ CFU/mL (low inoculum) at the beginning of incubation. As a result, *E. coli* (Figure 1A) was able to grow in the PM 1 (fresh fibers, bleached) and PM 3 (100% recycled fibers) media and no growth but a decrease in the number of bacteria was seen for PM 2 (50% recycled fibers) and PM 4 (fresh fibers, unbleached). Thereby, *E. coli* reached a maximum bacterial count of 8.6 log_10_ CFU/mL in PM 3 within 3 days and 7.0 log_10_ CFU/mL in PM 1 after 7 days. In contrast, *S. aureus* decreased in all tested media and no growth was observed (Figure 1B). *Bacillus cereus* could only grow in the 100% recycled fibers medium PM 3 reaching a maximum of 6.1 log_10_ CFU/mL (Figure 1C). Furthermore, it remained at stable bacterial counts in PM 1, while decreasing in PM 2 and PM 4. In addition to growth, survival was evaluated at an initially added bacterial level of 5 to 7 log_10_ CFU/mL (high inoculum) to better visualize a potential decrease. Growth of *E. coli* even continued to be observed in PM 3 at high inoculation levels up to 9.9 log_10_ CFU/mL (Figure 1D). No significant changes in bacterial counts were observed in PM 1 and PM 2 suggesting stable survival in these media. In PM 4 however, *E. coli* could not sustain and was completely eliminated within 2 days. No survival was observed for *S. aureus* in any of the media tested (Figure 1E), which corresponds to the observations in the growth experiments with lower spike-in levels. Nevertheless, incubation in PM3 resulted in longer survival and a slower decline for *S. aureus*. Similar to the growth results, *B. cereus* was able to survive in PM 1 and PM 3 without major changes in bacterial numbers (Figure 1F), but decreased in PM 2 and PM 4 without disappearing completely. As a control, the bacterial counts of each inoculum were determined prior to inoculation of the media. For each bacterial species, correct inoculations were observed with deviations of less than 1-log_10_ in the final medium to the inoculum. After each growth and survival experiment, the presence of the inoculated species was also correctly confirmed by MALDI-TOF identification.

### 3.2. Identification of packaging material isolates

To investigate the interaction of typical packaging material bacteria with different types of packaging materials, two isolates from PM 3 (100% recycled fibers) were collected, identified on species level and then tested for their growth and survival capacities. The MALDI-TOF identification revealed *Bacillus firmus* and *Bacillus circulans* for the two isolates, which was confirmed by 16S rRNA gene sequencing giving *Cytobacillus* (*C*.) *firmus* and *Nialla* (*N*.) *circulans* as a result. *Cytobacillus firmus* and *Niallia circulans* are both Gram-positive, rod-shaped endospore forming bacteria from the Firmicutes. For both species, new genera were recently proposed separating them from the genus *Bacillus* (Gupta et al., 2020; Patel and Gupta, 2020).

### 3.3. Growth and survival studies on packaging material isolates

**Figure 2:**
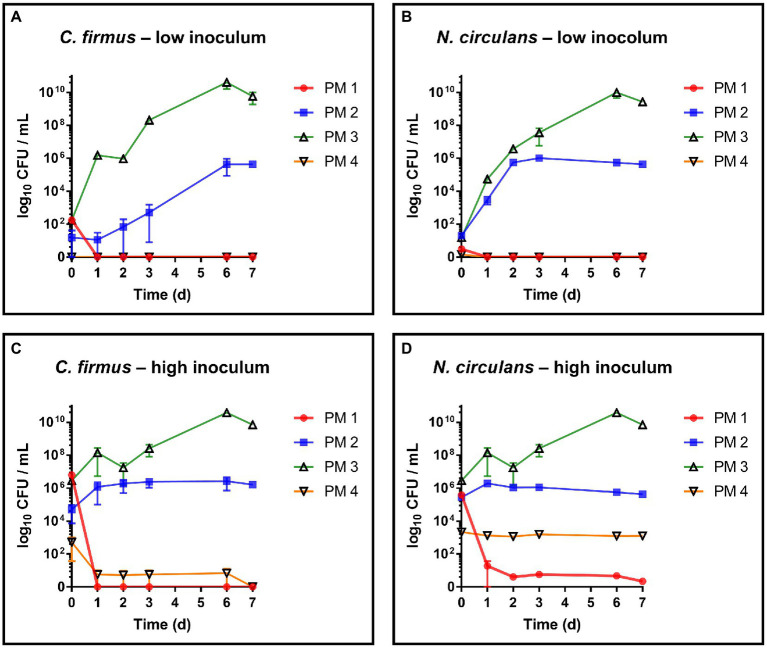
Evaluation of growth and survival of packaging material isolates. Growth and survival were evaluated over time in liquid media solely consisting homogenized packaging materials. PM 1 (red) consists of bleached fresh fibers, PM 2 (blue) consists of 50% recycled fibers, PM 3 (green) consists of 100% recycled fibers, and PM 4 (yellow) consists of unbleached fresh fibers. **(A)** *Cytobacillus firmus* (*C. firmus*), low inoculum (1–3 log_10_ CFU/mL), **(B)** *Niallia circulans* (*N. circulans*), low inoculum, **(C)** *Cytobacillus firmus* (*C. firmus*), high inoculum (5–7 log_10_ CFU/mL), **(D)** *Niallia circulans* (*N. circulans*), high inoculum. All experiments were performed in biological duplicates. The mean with range is plotted.

**Figure 3:**
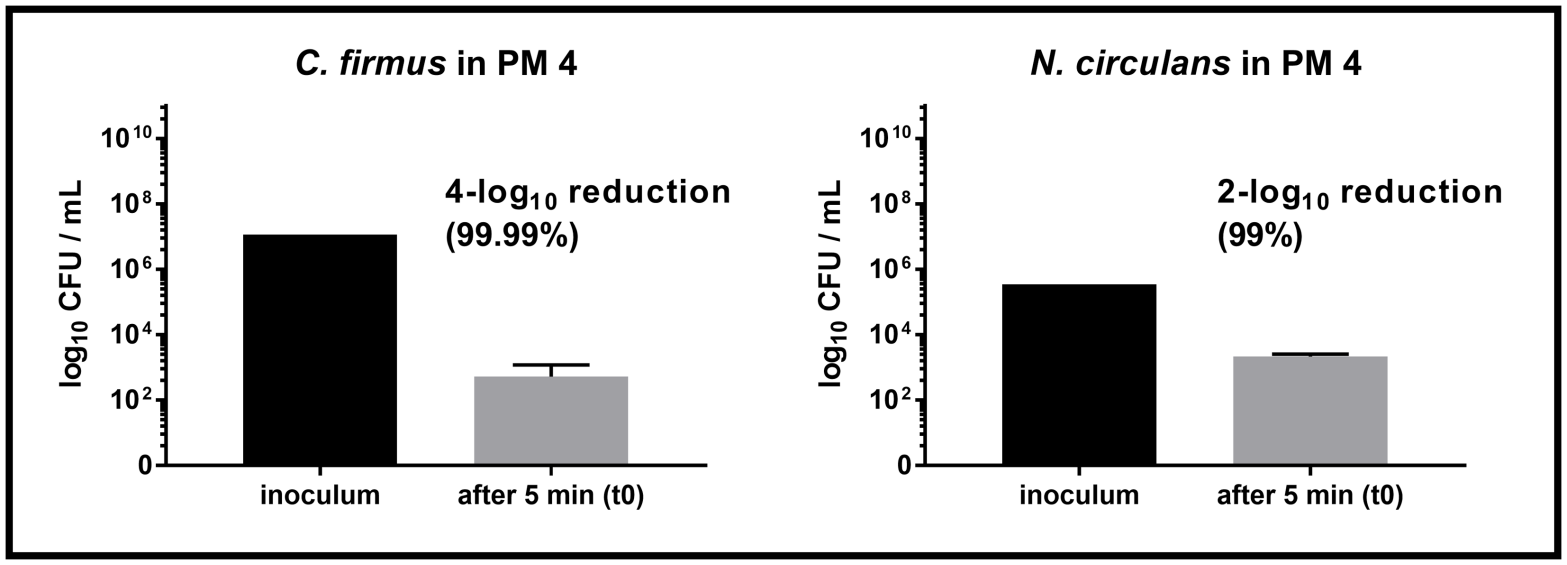
Comparison of inoculum and recovery in PM4. Comparison of the inoculum (initially spiked-in) and bacterial count after 5 min (t0) in the packaging material medium PM 4. Substantial reduction was only observed for *Cytobacillus firmus* (*C. firmus*, left) and *Niallia circulans* (*N. circulans*, right). Inoculum was prepared once (initially spiked-in). Inoculation of PM 4 and CFU counting after 5 min (t0) was done in biological duplicates. The mean with standard deviation is plotted.

Both strains, *C. firmus* and *N. circulans*, were isolated from PM 3 and showed strong growth in the PM 3 based medium consisting exclusively of recycled fibers (Figures 2A,B). When added to the PM 3 medium at low initial spike-in counts (low inoculum) *C. firmus* and *N. circulans* reached maxima of 10.8 log_10_ CFU/mL and 10.2 log_10_ CFU/mL, respectively, after 6 days of incubation, which clearly exceeded the growth capacities of tested *E. coli* and *B. cereus* strains in this packaging material medium. Moreover, microbial growth of *C. firmus* and *N. circulans* was also observed in the PM 2 medium composed of 50% recycled fibers, reaching up to 5.6 log_10_ CFU/mL after 6 days and 6.0 log_10_ CFU/mL after 3 days of incubation, respectively. No growth could be observed for *C. firmus* and *N. circulans* in PM 1 and PM 4 media, both consisting of fresh fibers, which may indicate a specific interaction of these two isolates with packaging materials containing recycled fibers. Interestingly, in PM 4, *C. firmus* was already undetectable directly after inoculation at time point 0, which pointed toward an antimicrobial effect that emanates from this packaging material type. The inoculum control revealed a bacterial count of 3.2 log_10_ CFU/mL present in the medium after inoculation, thus, a 3-log_10_ reduction (99.9%) of *C. firmus* was observed in PM 4. This immediate antimicrobial effect in the PM 4 medium could also be seen for *N. circulans*, which was reduced from 1.5 log_10_ CFU/mL after inoculation to a mean count of below 0 log_10_ CFU/mL (n = 2) at time point 0, followed by a complete elimination. When added to the PM 3 medium at high initial spike-in counts for survival monitoring (high inoculum), *C. firmus* und *N. circulans* reached comparable counts of CFU/mL as in the growth experiments (Figures 2C,D). Therefore, it confirms the extend of growth potential. Reduced growth of *C. firmus* and *N. circulans* was observed in PM 2 medium, confirming the extent of growth potential in this medium determined by the previous growth experiment. Unlike the food contaminants *E. coli* and *B. cereus*, the PM 3 isolates *C. firmus* and *N. circulans* could not sustain in PM 1 consisting of fresh fibers. While *C. firmus* has already disappeared completely after 1 day of incubation, *N. circulans* decreased notably but remained detectable in the medium for up to 7 days at levels below 1 log_10_ CFU/mL. Incubation in PM 4 medium resulted in a reduction of *C. firmus*, but it was still detectable at levels below 1 log_10_ CFU/mL (Figure 2C). As for the growth experiment, an immediate antimicrobial effect was seen if comparing the inoculation of PM 4 to 7.1 log_10_ CFU/mL final concentration with the detected number 3 log_10_ CFU/mL (*n* = 2) at time point 0 (after 5 min), which corresponds to a 4-log_10_ reduction (99.99%) of inoculated bacteria (Figure 3). Although a continuous decrease over time could not be observed for *N. circulans* in PM 4 (Figure 2D), a discrepancy between inoculated bacteria and detected bacteria after 5 min was present. The inoculated bacteria of 5.5 log_10_ CFU/mL final concentration in the medium were reduced to 3.3 log_10_ CFU/mL, corresponding to a 2-log_10_ reduction (99%) (Figure 3). The low-level survival of *C. firmus* and *N. circulans* throughout the incubation period resembled the observations for *B. cereus* in PM 2 and PM 4 (Figure 1F), suggesting a connection to the presence of endospores. Correct inoculations with *C. firmus* and *N. circulans* were observed for PM 1 and PM 2 with deviations of less than 1-log_10_. Inoculation of PM 3 tended to be more unstable which resulted in deviations of less than 2-log_10_ (Data not shown). After each growth and survival experiment, the presence of the inoculated species was also correctly confirmed by MALDI-TOF identification.

### 3.4. Germination and growth capabilities of *Bacillus subtilis* and *Bacillus cereus* endospores

**Figure 4:**
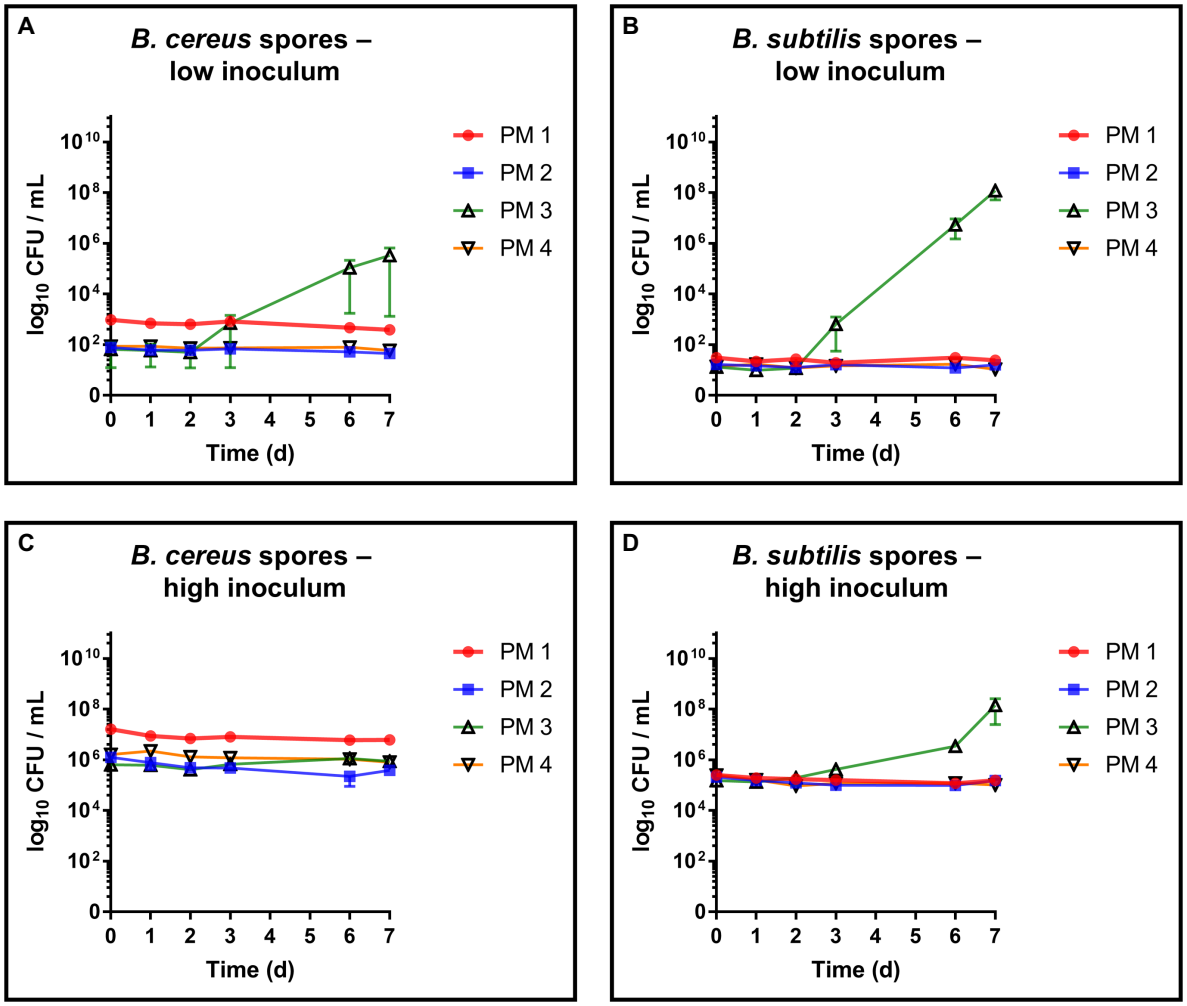
Evaluation of spore germination and growth capabilities. Spore germination and growth capabilities of *B. cereus* and *B. subtilis* endospores were evaluated over time in different liquid media solely consisting homogenized packaging materials. PM 1 (red) consists of bleached fresh fibers, PM 2 (blue) consists of 50% recycled fibers, PM 3 (green) consists of 100% recycled fibers, and PM 4 (yellow) consists of unbleached fresh fibers. **(A)** *Bacillus cereus* endospores, low inoculum (1–3 log_10_ CFU/mL), **(B)** *Bacillus subtilis* endospores, low inoculum, **(C)** *Bacillus cereus* endospores, high inoculum (5–7 log_10_ CFU/mL), and **(D)** *Bacillus subtilis* endospores, high inoculum. All experiments were performed in biological duplicates. The mean with range is plotted.

**Figure 5:**
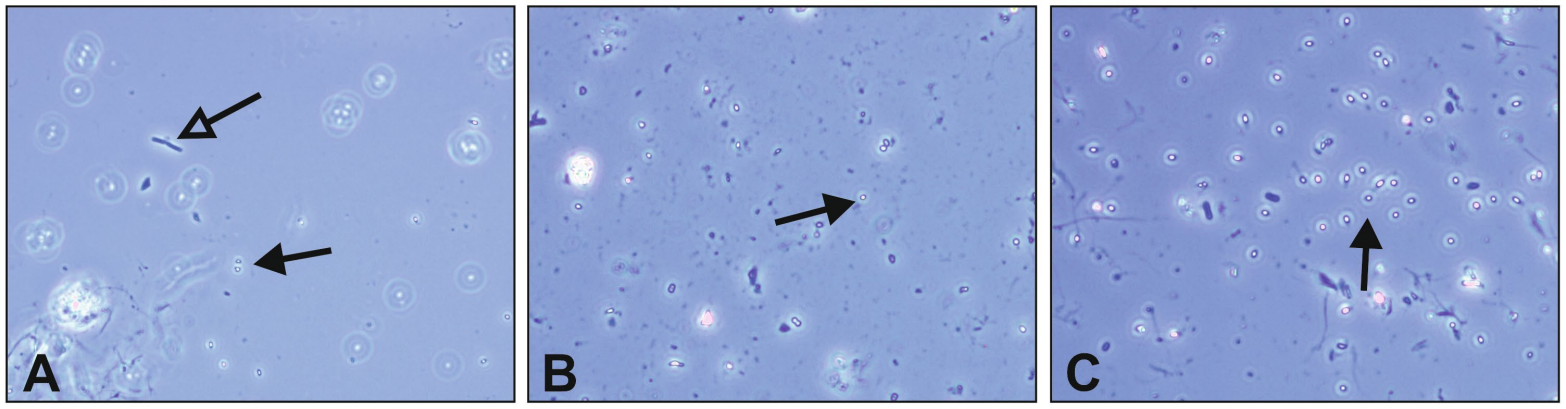
Microscopic evaluation of *Bacillus cereus* endospore germination. Endospore germination was evaluated in liquid growth media solely based on different packaging materials. Spore germination was visually assessed in pelleted media using phase-contrast microscopy after 7 days of incubation at 30°C. Dormant bacterial spores are indicated with black arrows, while vital cells emerging from germination are indicated with empty arrows. **(A)** *Bacillus cereus* spores and vital bacterial cell in PM 1, **(B)** *Bacillus cereus* spores in PM 2, and **(C)** *Bacillus cereus* spores in PM 4. The packaging material medium PM 3 was excluded due to pronounced bacterial growth during incubation, making germination evaluation redundant.

The germination capacity of free bacterial endospores was assessed by including a commercial *B. subtilis* subsp. *spizizenii* spore suspension as well as a laboratory made spore suspension of *B. cereus* into our growth and survival experiments. For both strains, spore germination and subsequent growth was exclusively observed in PM 3 medium (100% recycled fibers) (Figures 4A,B). This is in accordance with the results from the growth experiments with vegetative *B. cereus*, whereas lower bacterial counts were detected with a maximum of 5.8 log_10_ CFU/mL (Figure 4A). In sharp contrast to vegetative *B. cereus* cells, no decrease was observed in PM 2 and PM 4, pointing to the increased stability of bacterial endospores compared to vegetative cells. Nevertheless, neither PM 1, PM 2 nor PM 4 allowed microbial growth when bacterial endospores were added. The inoculation with *B. subtilis* endospores resulted in comparable observations as for *B. cereus* endospores (Figure 4B). Growth of *B. subtilis* was exclusively observed in PM 3 medium, but at both low and high bacterial inoculum up to 8.3 log_10_ CFU/mL (Figure 4C) and 8.4 log_10_ CFU/mL (Figure 4D), respectively. Neither growth nor decrease of *B. subtilis* was seen in PM 1, PM 2 and PM 4 suggesting only a stable survival of dormant bacterial endospores (Figures 4C,D). This suggestion is supported by the correct identification of *B. subtilis* subsp. *spizizenii* and *B. cereus* using MALDI-TOF after completion of the experiments, as well as the generally correct inoculation with *B. subtilis* and *B. cereus* spores with deviations of less than 1-log_10_. A deviation of more than 1-log_10_ was observed for the growth experiment inoculation of PM 3 with *B. cereus* spores (Data not shown), however, without major effects due to growth in PM 3 medium. Microscopic examination of pelleted growth media confirmed the limited potential of bacterial spores to germinate in PM 1, PM 2 and PM 4 (Figure 5). Germination of *B. cereus* spores was only observed very occasionally in PM 1 (Figure 5A), whereas no germination of *B. subtilis* was found in PM 1, PM 2 and PM 4.

### 3.5. Bacterial digestion of carboxymethyl cellulose and xylan

**Figure 6:**
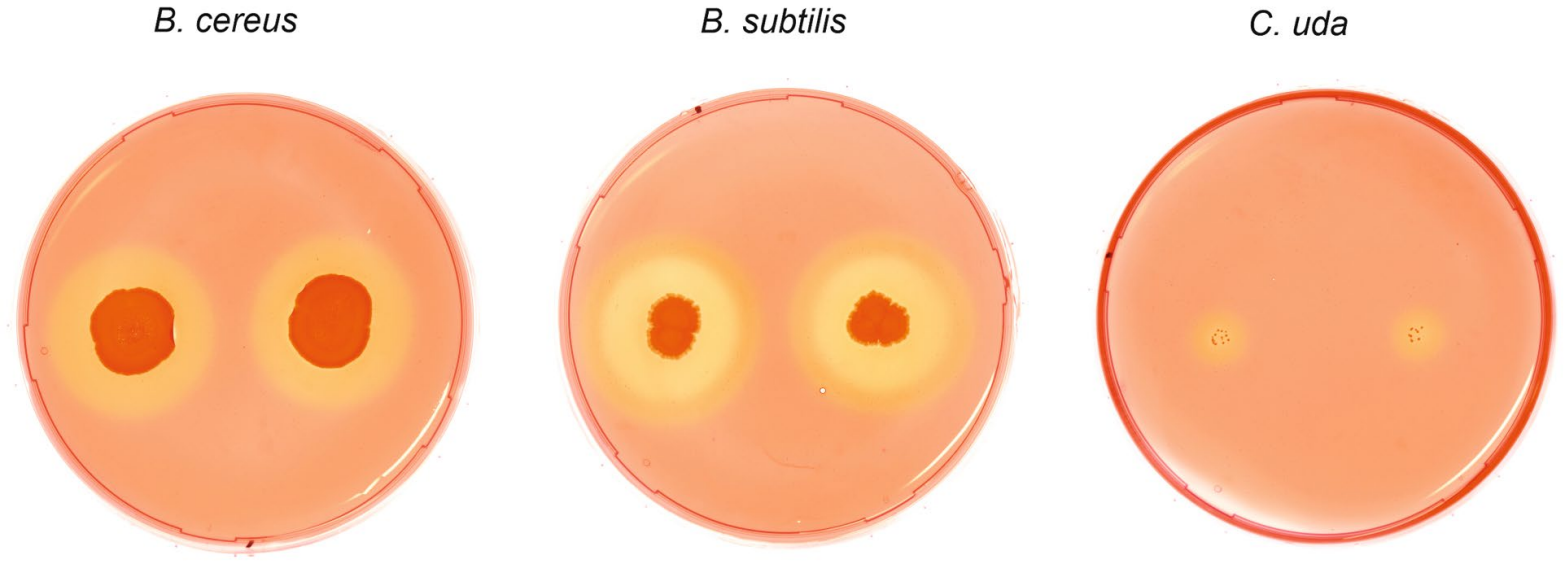
Cellulose degrading phenotypes of *Bacillus cereus*, *Bacillus subtilis* and *Cellulomonas uda.* Spot inoculation of *B. cereus*, *B. subtilis* and *C. uda* (control) on CMC medium A. Plates were stained with 0.1% Congo red solution to highlight CMC degradation indicated by clear halo formation. Two halos were observed for *B*. *subtilis*.

To investigate cellulolytic and hemicellulolytic properties, all strains were plated on two different media containing sodium carboxymethyl cellulose (CMC medium A and B) as well as one xylan-containing medium. Regarding the digestion of CMC, bacterial growth was only observed on medium A, whereas no colonies were detected on medium B indicating growth inhibition emanating from Remazol Brilliant Blue (Table 3). After spot inoculation and staining of CMC medium A, halos were observed for *B. cereus* and *B. subtilis* indicating bacterial cellulose degradation, including a double halo for *B. subtilis* (Figure 6). The evaluation of halo zones revealed large activity for *B. subtilis* (8.3 mm inner halo and 12.3 mm outer halo) and *B. cereus* (7.7 mm) compared to the positive control *Cellulomonas uda* (2.7 mm). However, bacterial growth of *C. uda* was noticeably slower, which resulted in very small colonies and compared to the large colonies of *B. cereus* and *B. subtilis*. Moreover, *C. firmus* turned out to be a more fastidious species and cellulolytic properties could not be assessed on CMC medium A. However, incubation on TSA supplemented with CMC resulted in bacterial growth, but no signs of cellulose degradation, while the control strain *C. uda* showed degradation on this medium. On xylan medium, all strains except for *C. firmus* were able to grow. After Congo red stain, clear halos were visible only for *B. subtilis* (16.7 mm) as well as for the positive control *C. uda* (8.7 mm) indicating digestion of the hemicellulose xylan. Incubation on control media without supplements (CMC or xylan) did not lead to halo formation on the respective media. Based on the results from previous growth experiments in packaging material media, no association could be established between the cellulolytic or xylanolytic properties and the specific microbial growth in packaging material.

**Table 3:** Phenotypes of tested bacterial strains on different CMC media and xylan medium.

Growth medium	*E. coli*	*S. aureus*	*B. cereus*	*C. firmus*	*N. circulans*	*B. subtilis*	*C. uda*
CMC medium A	(+)	(+)	7.7 mm	(−)	(+)	8.3 mm (12.3 mm)	2.7 mm
CMC medium B	(−)	(−)	(−)	(−)	(−)	(−)	(−)
Xylan medium	(+)	(+)	(+)	(−)	(+)	16.7 mm	8.7 mm

If visible colonies with clear halos were detected, the sizes of the halos are shown in mm. If a second, outer halo was visible, the size in mm is given in brackets. (+) indicates visible colonies without any halo on the respective growth medium, while (−) indicates no visible microbial growth.

### 3.6. Influence of the pH value of the packaging material on bacterial growth

**Figure 7:**
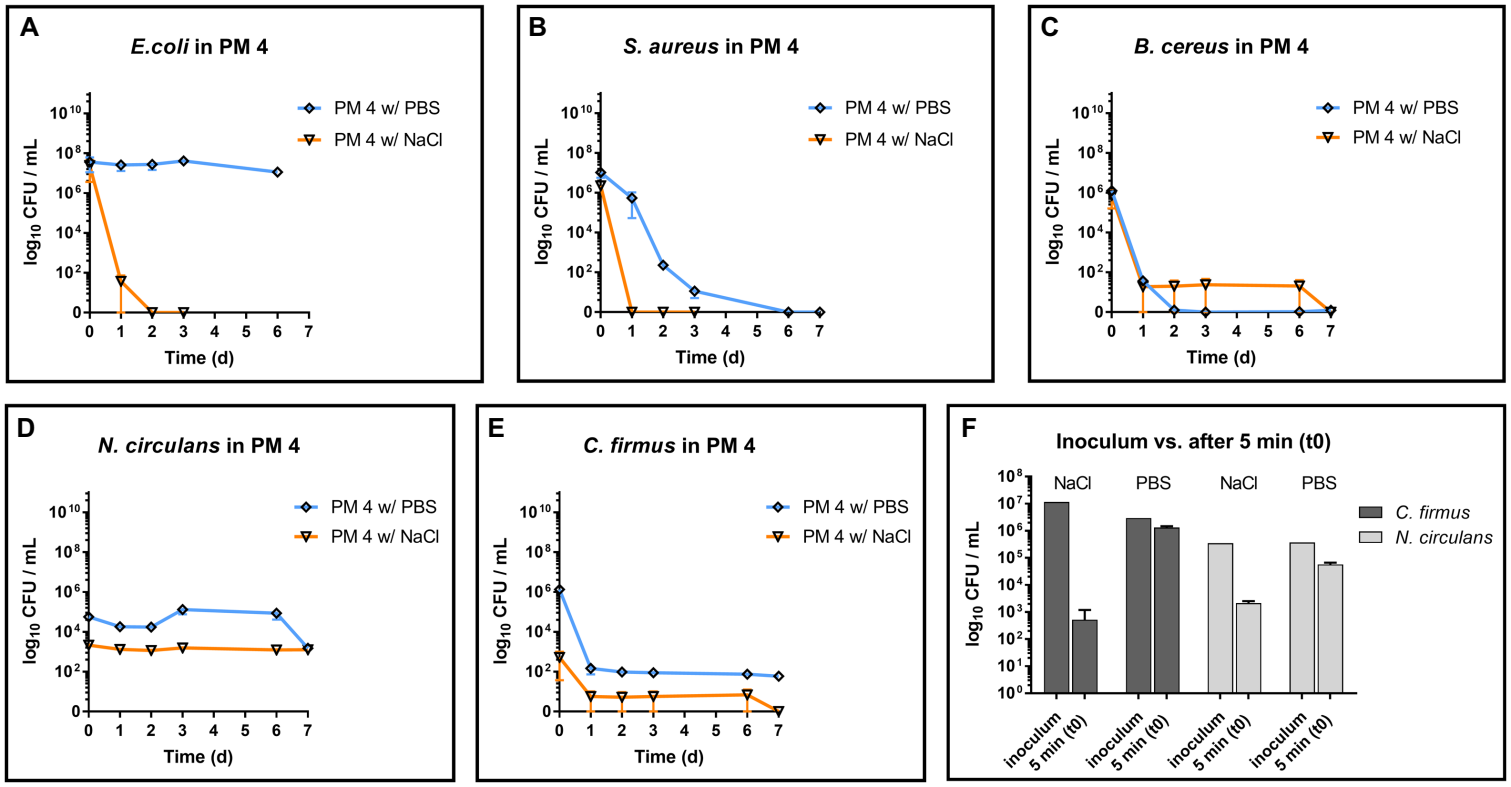
Influence of PBS and saline solution on microbial survival in PM 4. Comparison of microbial survival in PM 4 growth media based on PBS or saline solution (0.9% NaCl). Packaging material PM 4 was homogenized either with PBS (orange, pH = 7.40) or with saline solution (blue, pH = 4.46) and inoculated with 5–7 log_10_ CFU/mL for survival monitoring of the test strains **(A–E)**. The mean with range is plotted. Additionally, the prepared inoculum and the bacterial counts after 5 min (t0) in the packaging material medium PM 4 were compared for *C. firmus* and *N. circulans* **(F)**. The mean with standard deviation is plotted.

Although the exact compositions of the packaging materials are not known, a potential factor for observed antimicrobial activity in PM 4 could be determined by pH measurement of the media. The pH for growth media made of PM 1, PM 2 and PM 3 were 6.32, 7.98 and 8.21, respectively, and thus did not deviate widely from neutral pH. In contrast, PM 4 medium showed a considerably lower pH of 4.46, which provides harsh conditions for microbial growth. In order to dissect the detrimental effects of pH in homogenized PM 4, the sample was additionally homogenized with PBS to maintain a neutral pH of 7.40 and inoculated with the test strains for growth monitoring. Growth experiments revealed a stable survival of *E. coli* instead of rapid decrease (Figure 7A) and prolonged survival of *S. aureus* in PM 4 with PBS (Figure 7B). For members of the Bacillaceae (*B. cereus*, *N. circulans*, and *C. firmus*) the stabilizing effect of buffered growth medium could not be confirmed as the decrease of bacterial counts did not change notably compared to PM 4 with 0.9% NaCl (saline) solution (Figures 7C–E). However, the instant reduction of *C. firmus* and *N. circulans* in PM 4 homogenized with saline solution was not observed when PBS was used, giving evidence for the immediate effect of the pH on bacterial survival (Figure 7F). Furthermore, pH did not change significantly over incubation period in bacterial cultures of the Bacillaceae contradicting pH as the sole factor for antimicrobial activity.

## 4. Discussion

The awareness of food safety continues to grow and, as a result, there is a demand for appropriate food packaging that ensures the microbial integrity of food. Fiber-based packaging materials are widely used as primary food packaging (Deshwal et al., 2019) e.g. as bags for baked goods, pizza boxes, fast food containers, and especially cardboard crates for fruits and vegetables, as well as for secondary and tertiary food packaging applications. However, the effects of bacterial growth within these packaging materials have been scarcely studied. The curious lack of information regarding the microbial integrity of fiber-based packaging materials compared to other packaging systems has already been stated (Brandwein et al., 2016). A recent study investigating storage effects of fiber-based food packaging products on the microbial load reported significant reductions after 6 months of storage at 4°C (Zaidi et al., 2022). However, this long-term study lacked in more comprehensive investigations such as growth promoting temperatures, spiking with external microorganisms, and different time points. Our unique study contributes to fulfill this gap of knowledge by investigating the growth and survival of different species in fiber-based packaging materials, especially since all the packaging materials included are actually used in the food packaging sector (Table 1). These materials generally contain low quantities of bacteria per gram (Hladíková et al., 2015; Maitz et al., 2022), also supporting the need for growth studies in terms of food safety. Furthermore, our findings show possibilities to improve product safety but also storability and shelf life of both, packaged food and packaging material itself. It was suggested that certain packaging types select for and enrich certain microorganisms (Brandwein et al., 2016) which has been unequivocally confirmed by our results. Furthermore, we could show that physicochemical interactions with fiber-based packaging materials contribute to the thriving environment for bacteria, rather than surface adhesion properties or the three dimensional cellulose fiber networks. The superiority of fiber-based packaging materials to plastic packaging regarding cross-contamination could already been shown (Siroli et al., 2017), but explained the observed antimicrobial effects only by the physical structure of the porous fiber material. In contrast, we disintegrated the packaging material and therefore provided optimal growth conditions for bacteria including appropriate growth temperature, shaking in Erlenmeyer flasks and optimal water availability (water activity of 1.0). Thus, our growth-favoring model appears to be more suitable to study metabolic and chemical interactions of microorganisms with packaging materials, but disregarding the physical structures. Only reduced shaking to 50 rpm during incubation was necessary to prevent floc formation, guaranteeing maximal access to fibers and other compounds at the expense of not maximizing oxygen supply. Although in everyday food storage water activities above 0.9 and temperatures above 30°C are rarely reached, the present study investigated the basic principles of growth of different microorganisms in fiber-based packaging materials in very growth promoting conditions. Since a potential contact with liquids, e.g., rain, condensation, should not be neglected and appropriate storage temperatures might fail, this work gives a fundamental insight into the interaction of bacteria with the packaging material itself for a general risk assessment. In general it has long been known that microorganisms can decompose fiber-based materials (Ntaikou et al., 2009), which is a key aspect for an ecological and sustainable packaging material. Our study could reveal that microbial growth in the fiber-based materials is possible, but it depends on highly specific interactions of certain microorganisms with certain types of packaging material. Just because a species can grow in one packaging material does not imply it will grow in another due to the vast variety of different packaging types. Interestingly, *E. coli* was the only species able to grow in a bleached fresh fiber medium. The isolates *C. firmus* and *N. circulans*, on the other side, could only grow in two materials that contained recycled fibers, and growth of *B. cereus* was restricted to a material composed entirely of recycled fibers. These three species, belonging to the Bacillaceae, are typical packaging inherent bacteria (Suihko et al., 2004; Lalande et al., 2014; Schmid et al., 2021) and appeared to be specifically adapted to the composition of the recycled fibers materials. Evaluation of spore germination is essential, since endospore forming bacteria comprise the major part of packaging inherent bacteria (Väisänen et al., 1991; Suihko et al., 2004). As a matter of fact, these aerobic mesophilic spore-forming bacteria have no medical significance in most cases. From an industrial standpoint, however, germination and subsequent growth of packaging inherent bacteria could affect the product quality. Here again, spore germination and growth was associated with the secondary food packaging material made of 100% recycled fibers. This underlines the increased microbial integrity of fresh fiber products that come into direct contact with food compared to recycled packaging materials. This is also supported by the fact that increased recycling content correlates with increased bacterial load (Hladíková et al., 2015). The actual role of the fiber type, however, remains ambiguous, since manufacturing of recycled packaging materials also involves numerous additives and fillers such as starch and calcium carbonate (Hubbe and Gill, 2016). Unfortunately, the industry partners did not disclose the exact ingredients of the packaging materials only giving fiber-type and current application in the food packaging sector. Therefore, upcoming research should focus on the identification and the actual role of additives from a microbiological point of view. Furthermore, the negligible influence of the fibers is supported by the divergent cellulolytic and xylanolytic phenotypes of the tested bacteria, which did not allow any correlation with their growth potential. Since cellulose fibers are an integral part of fiber-based packaging materials, they can be denied a crucial role as a source of nutrients. The same applies to hemicelluloses, which are heteropolymers consisting of various sugar monomers. They are usually removed to varying degrees during the manufacturing of packaging materials and are also known as nutrients for microorganisms (Wedekind et al., 1988; Hu et al., 2013). However, the digestion of xylan was limited to *B. subtilis* indicating that hemicellulose digestion may not be a major contributor to microbial growth. Still, a hemicellulose contribution to microbial growth cannot be ruled out completely due to the exceptionally diverse class of hemicelluloses. More research on bacterial degradation of different hemicelluloses would be required, especially within complex materials. Methodologically, the specificity of CMC agar-based assays has been doubted (Johnsen and Krause, 2014). Nevertheless, it proved to be a valid screening tool, and we were able to demonstrate the independence of microbial growth from cellulolytic enzymes. Generally, the food packaging sector constantly strives for novel packaging strategies to extend shelf life, minimize cross-contamination and control microbial growth. This led to the continuous development of newly functionalized biomaterials, thin films and coatings to achieve antimicrobial food packaging. Novel approaches in active food packaging often use CMC, chitosan or poly lactic acid films, which are coupled with bioactive components such as ZnO, Ar or TiO_2_ nanoparticles, or quaternary ammonium compounds (Li et al., 2017, 2018). Despite their antimicrobial effectiveness, these functionalization strategies are associated with high costs and product safety has hardly been evaluated. There have been only few studies evaluating the interaction of microorganisms with already established food packaging materials (Brandwein et al., 2016; Siroli et al., 2017). A more detailed investigation of these materials can contribute to finding the appropriate packaging material for different applications from a microbiological perspective. Furthermore, it can reveal new targets for potential functionalization and elucidate already existing properties typical for active food packaging, such as the prevention of microbial growth. In our study, we could observe the strongest growth using the packaging material consisting exclusively of recycled fibers (PM 3). However, according to the manufacturer, this packaging material is not intended for direct contact with food, so the risk of cross-contamination of food is not possible. On the other side, all packaging materials intended for direct food contact prevented the growth of *S. aureus* and *B. cereus*. Based on these findings, fiber-based food packaging materials can be developed to prevent microbial growth in the packaging itself. Nevertheless, food processing hygiene is much more crucial for food safety, than the negligible risk that emanates from the fiber-based packaging materials (Ekman et al., 2009). We also detected a low pH of 4.5, which resulted in reduced survival for all tested bacterial species in PM 4. Unfortunately, we were not able to find the cause of this low pH and possible sources are manifold. Nevertheless, lowering of the pH has been known for a long time and is still used in food preservation to prevent microbial growth. Taking into account the relevant standards for food packaging materials (European Parliament, 2004) and the technical requirements during production, adjustment of the pH could provide a cost-effective and consumer-safe functionalization strategy of already established packaging materials. The fact that buffered media did not compensate all growth inhibiting effects strongly suggests further antimicrobial compounds active in the packaging medium. This also supports the need for further investigation of fiber-based packaging materials to find cost-effective and sustainable solutions with active packaging properties. Evaluation of bacterial growth in packaging materials is essential for future risk assessment in the food packaging sector. Our study provides information on the growth capacities but it is limited in the mathematical description of distinct growth parameters, mostly due to the strongly diverging growth phenotypes with different lag, log and stationary phases for the test strain in the diverse growth media. The focus on qualitative growth assessment rather than quantitative growth analysis, along with the small sample size of packaging materials, also resulted in a limited statistical analysis of the data. Nevertheless, all experiments were performed in replicates and descriptive statistics are given. Based on the results of this study, upcoming research will therefore concentrate on the growth kinetics in fiber-based packaging materials, taking mathematical models for bacterial growth into account.

## Data availability statement

The datasets presented in this study can be found in online repositories. The names of the repository/repositories and accession number(s) can be found at: https://www.ncbi.nlm.nih.gov/genbank/, OP422519, OP422520.

## Author contributions

PS: conceptualization, methodology, data collection and analysis, validation, and writing – original draft. SM: conceptualization, and writing – review and editing. NP: methodology, data collection and analysis, and visualization. EK: methodology, data collection and analysis. SP: methodology, writing – review and editing. CK: funding acquisition, supervision, and writing – review and editing. All authors have approved the final version to be published. All authors are to be accountable for all aspects of the work in ensuring that questions related to the accuracy or integrity of any part of the work are appropriately investigated and resolved.

## Funding

This work has been funded by the Christian Doppler Society, Austria (CD-Laboratory for Mass Transport through Paper). The financial support of the Austrian Federal Ministry of Labour and Economy and the National Foundation for Research, Technology and Development, Austria is acknowledged.

## Conflict of interest

The authors declare that the research was conducted in the absence of any commercial or financial relationships that could be construed as a potential conflict of interest.

## Publisher’s note

All claims expressed in this article are solely those of the authors and do not necessarily represent those of their affiliated organizations, or those of the publisher, the editors and the reviewers. Any product that may be evaluated in this article, or claim that may be made by its manufacturer, is not guaranteed or endorsed by the publisher.
